# Rapid Liver Fibrosis Evaluation Using the UNet-ResNet50-32 × 4d Model in Magnetic Resonance Elastography: Retrospective Study

**DOI:** 10.2196/80351

**Published:** 2025-10-20

**Authors:** Pei-Yuan Su, Han-Jie Shih, Jia-Lang Xu

**Affiliations:** 1 Department of Internal Medicine, Division of Gastroenterology, Changhua Christian Hospital, Changhua, Taiwan; 2 Department of Post-Baccalaureate Medicine, College of Medicine, National Chung Hsing University, Taichung, Taiwan; 3 Department of Medical Imaging, Changhua Christian Hospital, Changhua, Taiwan; 4 Department of Applied Statistics, National Taichung University of Science and Technology, No. 129, Section 3, Sanmin Road, North District, Taichung, 404336, Taiwan, 886 4-2219-6076, 886 4-2219-6330

**Keywords:** liver fibrosis, image segmentation, MRI elastography, magnetic resonance elastography, automated segmentation, timely decision-making

## Abstract

**Background:**

Liver fibrosis is a pathological outcome of chronic liver injury and a hallmark of multiple chronic liver diseases. Magnetic resonance elastography (MRE) provides a non-invasive modality for evaluating the severity of liver fibrosis.

**Objective:**

This study aimed to develop and evaluate deep learning–based segmentation models for the automated assessment of liver fibrosis using MRE images, with a focus on comparing the performance of a conventional U-Net model and a UNet-ResNet50−32 × 4d architecture model.

**Methods:**

A retrospective analysis was conducted on 319 patients enrolled between January 2018 and December 2020. MRE images were processed and segmented using two U-Net–based models. Model performance was assessed through correlation coefficients, intersection over union (IoU), and additional segmentation metrics.

**Results:**

The UNet-ResNet50−32 × 4d model demonstrated strong agreement with ground truth annotations, achieving correlation coefficients of 0.952 in the training phase and 0.943 in the validation phase, along with an Dice score of 85.68%, confirming its high segmentation accuracy.

**Conclusions:**

The UNet-ResNet50−32 × 4d model exhibited robust performance and may serve as a reliable tool for the rapid and accurate assessment of liver fibrosis severity. The integration of automated segmentation into MRE analysis has the potential to improve clinical workflows and support timely decision-making in the management of chronic liver disease.

## Introduction

### Research Background and Motivations

Liver fibrosis is a progressive process that culminates in cirrhosis, a condition associated with severe complications such as ascites, esophageal varices, and hepatic encephalopathy, which significantly shorten life expectancy [1,2]. To better stratify disease severity and guide management, fibrosis is commonly staged into five categories (F0–F4). F0 represents the absence of fibrosis, while F1–F3 correspond to progressive but non-cirrhotic stages of chronic liver disease, during which timely intervention can slow or even reverse disease progression. F4 indicates established cirrhosis, marking a critical threshold where the risk of decompensation, hepatocellular carcinoma, and mortality rises sharply. This staging framework not only provides prognostic insights but also plays a pivotal role in therapeutic decision-making, surveillance strategies, and patient counseling. The progression of liver fibrosis is slow and takes years to progress from mild liver fibrosis to cirrhosis. If liver fibrosis is diagnosed early using quicker and more convenient tools, there is a chance to prevent further deterioration into cirrhosis [3]. Currently, there are many tools for diagnosing liver fibrosis, with liver biopsy being the most accurate. However, due to the risk of bleeding, it is less commonly used [4]. There are many non-invasive testing methods, such as ultrasound elastography and magnetic resonance elastography (MRE), which have a high capability for diagnosing liver fibrosis [5,6]. MRE requires manual circling of images for interpretative reading, which increases the time for doctors to interpret reports [7]. Therefore, using image segmentation technology could help reduce the time for interpreting MRE for liver fibrosis and improve accuracy.

Artificial intelligence (AI) has been widely investigated in the medical field, with numerous applications across disease prediction, diagnosis, and clinical decision support. For instance, computed tomography (CT) imaging has shown strong potential in predicting cholangiocarcinoma recurrence [8], while machine learning–based image analysis methods have been applied for diabetic foot evaluation [9]. Convolutional neural networks (CNNs) have been employed to predict clinical outcomes in patients with stroke [10], and a YOLOv8 model has demonstrated a high accuracy in early lung cancer detection [11]. Similarly, combining chest X-rays with clinical features has yielded favorable area under the curve performance in osteoporosis screening [12]. In health care operations, machine learning models have also been developed to predict emergency department patient flow, effectively estimating both hourly and daily visit volumes [13]. Moreover, clinical decision support systems have been explored for prenatal abnormality diagnosis and ultrasound applications, though such studies have yet to incorporate maternal or fetal data during pregnancy [14]. CNNs have been widely applied in liver tumor classification, while diverse architectures such as U-Net, UNet++, Residual Networks (ResNet), SegNet, and fully convolutional networks have been employed for semantic segmentation tasks [6,15-18]. However, despite this progress, only one previous study has reported the application of CNNs for MRE measurement [19]. By integrating the strengths of U-Net and ResNet, our Unet-ResNet model achieved superior segmentation performance and training stability, while maintaining strong agreement with manual evaluation.

Image segmentation represents another important domain of AI applications, whereby algorithms cluster elements of a similar nature into coherent segments [20]. This technique has been increasingly applied in medical imaging, including ultrasound imaging [21], CT scans [22], magnetic resonance imaging (MRI) [23], and X-ray imaging [24]. In liver-related applications, segmentation methods such as real-time liver ultrasound segmentation [17] can greatly assist physicians in diagnosis and treatment planning [25], while CT-based segmentation is useful for localizing liver tumors [26]. Advanced approaches using deep CNNs [27] and 3D deeply supervised networks [16] have further improved automated liver segmentation performance. Researchers have compared U-Net and V-Net architectures to assess their effectiveness in the segmentation of microcalcifications [28].

### Research Objectives

The aim of this study is to use image segmentation technology for the computational interpretation of MRE, enabling AI to automatically segment MRE images, accurately identify regions of interest, quantify liver fibrosis levels, and apply this approach to both training and validation cohorts. Early and reliable assessment of liver fibrosis is essential for timely clinical decision-making, yet conventional manual labeling of MRE images is time-consuming, operator-dependent, and prone to variability. To address these challenges, this study further investigates whether advanced architectures such as UNet-ResNet50−32 × 4d can achieve superior predictive performance compared with the traditional U-Net model.

## Methods

### Study Overview

**Figure 1.**
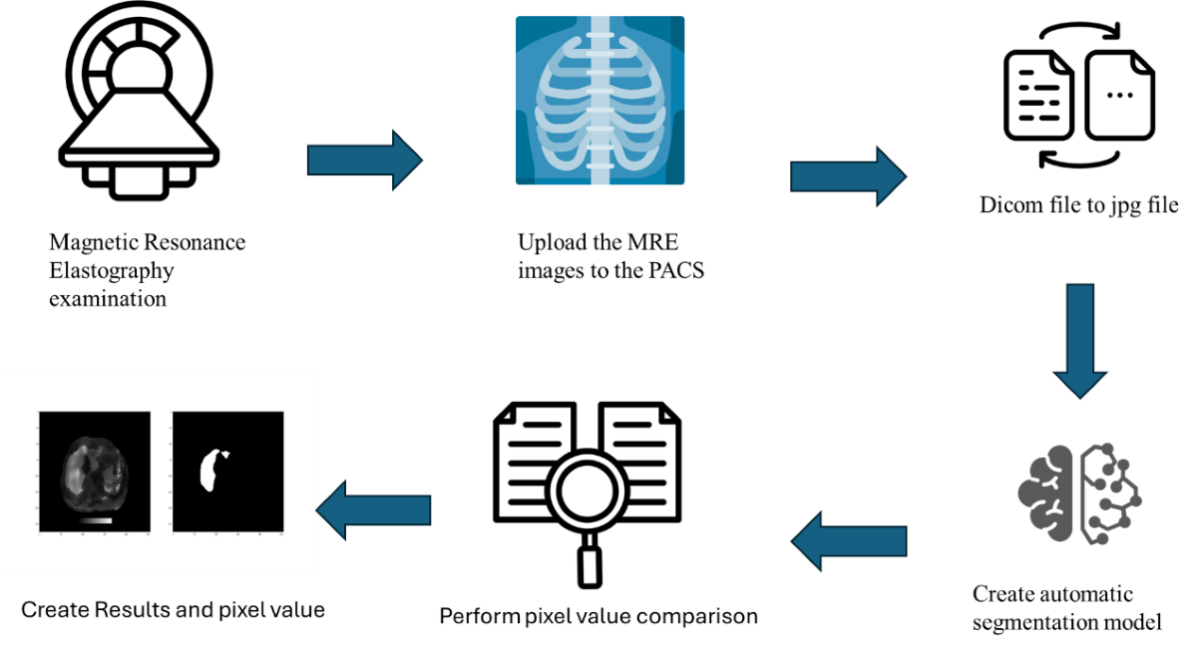
Overview of the automated workflow for liver fibrosis assessment using MRE confidence maps, illustrating image preprocessing, segmentation with the UNet-ResNet50−32×4d model, and quantitative analysis for clinical application. DICOM: Digital Imaging and Communications in Medicine; MRE: magnetic resonance elastography; PACS: picture archiving and communication system.

An overview of the automated workflow developed in this study is shown in Figure 1. This study incorporates an automated process in which patients undergo MRE examinations, and the imaging data are subsequently uploaded to the picture archiving and communication system. Once the upload is completed, the segmentation model is automatically triggered. During the validation phase, pixel values of the target regions are obtained directly from the Digital Imaging and Communications in Medicine (DICOM) images in the picture archiving and communication system, as interpreted by experienced radiologists. Since there are discrepancies between pixel values in DICOM format and those converted to JPEG, this study relies solely on the pixel values displayed in the original DICOM files for analysis. Due to discrepancies between pixel values in DICOM and JPEG formats, this study exclusively uses original DICOM pixel values for analysis. These values are automatically extracted using medical image processing libraries that read directly from the DICOM metadata and image matrix. After segmentation, the predicted masks and corresponding pixel values are overlaid on the original images and provided to radiologists to support clinical evaluation and decision.

### Ethical Considerations

The study was approved by the Institutional Review Board of Changhua Christian Hospital (approval no.: 210132). The Institutional Review Board waived the need for informed consent considering the retrospective nature of data collected. This study implemented stringent measures to protect the privacy of all participants by anonymizing all collected data to remove any personally identifiable information.

### Patients

This retrospective study collected patient data from January 2018 to December 2020. Patients were eligible if they underwent MRI for clinical indications and MRE was performed as part of the MRI protocol. Additional inclusion criteria were age greater than 20 years and the availability of demographic information (age and gender). Exclusion criteria consisted of incomplete imaging or missing demographic data. Based on these criteria, a total of 320 patients were initially identified, and 1 patient was excluded due to incomplete data, resulting in a final cohort of 319 patients included in the analysis. To ensure the broad applicability and usability of the proposed model, no further stratification was made according to the presence of chronic liver disease or fibrosis.

### MRE

MRI was performed with a 1.5-Tesla Aera magnet system by Siemens AG, equipped with a 16-channel phased-array body coil. The process involved a specialized MRE setup, integral to which was an acoustic driver system by Resoundant. The technical details of the MRE imaging sequence were as follows: a repetition time of 50 ms and an echo time of 22.7 ms, a flip angle of 25 degrees, and a bandwidth of 260 Hz/pixel. Additionally, the sequence settings included a hydrogen resonance frequency of 63.5 MHz, an acquisition matrix of 256 × 64, a section thickness of 5 mm, and a field of view of 400 × 400 mm^2^. For each patient, four to five confidence maps were automatically generated and post-processed directly on the MRI scanner workstation using the integrated MRE software on the Siemens Syngo MR VE11 system (MAGNETOM Aera, Skyra, and Avantofit; Siemens Healthineers), demonstrating adequate wave amplitudes in specific regions. Manual liver stiffness measurements were performed by an expert, who delineated the regions of interest on the confidence maps and calculated the stiffness values [29]. The stage of liver fibrosis was classified into four categories based on criteria developed at the same institute as this study [30]. Significant fibrosis was defined as stage F2 according to the METAVIR scoring system, corresponding to MRE values ≥2.8 kPa.

### MRE Image Labeling

For this study, the model training was conducted using data from 92 patients, all of whom had confidence maps generated from MRE. The annotations for these training images were meticulously created by a gastroenterologist, ensuring the reliability and precision of the ground truth used in model development.

### Statistical Analysis

The *t* test was used for comparison of continues variables of baseline characteristics. Continuous variables were showed as mean (SD). The Pearson correlation coefficient was performed to measure the correlation of the two MRE measurements by manual and automatic methods. All statistical analysis were performed on SPSS version 22.0 (IBM Corp.), with two-tailed *P* values <.05 indicating statistical significance.

### Model Training

The MRE images used in this study were acquired with a window center of 400 and a window width of 800, with an original resolution of 256×204 pixels. Since convolutional operations in the U-Net architecture require adequate spatial information for effective multi-scale feature extraction, all images were resized to 512×512 pixels using bilinear interpolation prior to model training. This resizing step was performed to preserve structural details and improve segmentation accuracy. For liver fibrosis segmentation, a hybrid deep learning model was implemented by combining U-Net with a ResNet50−32 × 4d encoder, leveraging its capacity to extract multi-scale contextual features while maintaining spatial resolution. Model optimization aimed to enhance both training efficiency and segmentation performance through systematic adjustment of key hyperparameters, with a comprehensive tuning strategy applied across a range of values, as summarized in Table 1. To address the pixel-level classification task, the model employed the Binary Cross-Entropy with Logits Loss as shown in equation (1).


(1) $$L=-\frac{1}{N}\sum_{i=1}^{N}\left[y_{i}\log(p_{i})+(1-y_{i})\log(1-p_{i})\right]$$

 Where $y_{i}$ denotes the true label of the i-th sample, $p_{i}$ represents the predicted probability that the sample belongs to the positive class, and N indicates the total number of samples.

**Table 1.** Hyperparameter tuning strategy and optimal settings for the model.

Hyperparameter	Values
Batch size	32
Loss	Binary Cross-Entropy with Logits Loss
Optimizer	Stochastic Gradient Descent
Learning rate	3×10⁻⁶–3×10⁻²
Weight decay	0‐0.01
Momentum	0.80‐0.99

## Results

### Cohort Characteristics

A total of 319 patients were enrolled in the study and divided into two cohorts, with 91 patients assigned to the training group and 228 patients allocated to the testing group. The baseline characteristics of the two cohorts are summarized in Table 2. The mean (SD) age was 57.2 (12.4) years in the training group and 52.6 (12.3) years in the testing group. There were no statistically significant differences in age, gender distribution, height, weight, or BMI between the two groups, as all *P* values were greater than .05. Similarly, the mean (SD) MRE stiffness values obtained by manual measurement were 4.51 (2.85) kPa in the training group and 3.69 (2.26) kPa in the testing group, with a *P* value of .09. The mean (SD) automated measurement values were 4.04 (1.93) kPa in the training group and 3.69 (2.28) kPa in the testing group with a *P* value of .41, also showing no significant difference. In contrast, a significant difference was observed in the proportion of patients with clinically significant fibrosis stage equal to or greater than F2. Based on manual MRE assessment, 76% (69/91) of patients in the training group had fibrosis stage F2 or higher compared with 52% (118/228) in the testing group, with a *P* value of less than .001. Automated MRE analysis identified 75% (68/91) of patients in the training group and 50% (114/228) in the testing group with fibrosis stage F2 or higher, also with a *P* value of less than .001. These findings confirm the consistency between manual and automated staging, while indicating that the prevalence of significant fibrosis was higher in the training cohort.

**Table 2.** The baseline characteristics of the training group and testing group.

Characteristics	Training group (n=91)	Testing group (n=228)	*P* value
Age, years, mean (SD)	57.2 (12.4)	52.6 (12.3)	.77
Gender, male (%)	48 (53)	137 (60)	.23
Height, m, mean (SD)	1.62 (0.08)	1.65 (0.08)	.97
Weight, kg, mean (SD)	65.8 (13.2)	67 (12.5)	.89
BMI, kg/m^2^, mean (SD)	25.1 (4.2)	24.6 (3.7)	.21
MRE^a^ (manual), kPa, mean (SD)	4.51 (2.85)	3.69 (2.26)	.09
MRE (automatic), kPa, mean (SD)	4.04 (1.93)	3.69 (2.28)	.41
Fibrosis stage (≥ F2^b^) (manual), n (%)	69 (76)	118 (52)	<.001
Fibrosis stage (≥ F2^b^) (automatic), n (%)	68 (75)	114 (50)	<.001

a MRE: magnetic resonance elastography.

b Liver fibrosis severity was determined using MRE, with stage F2 defined as MRE ≥2.8 kPa [30].

### Automatic Labeling Process

**Figure 2.**
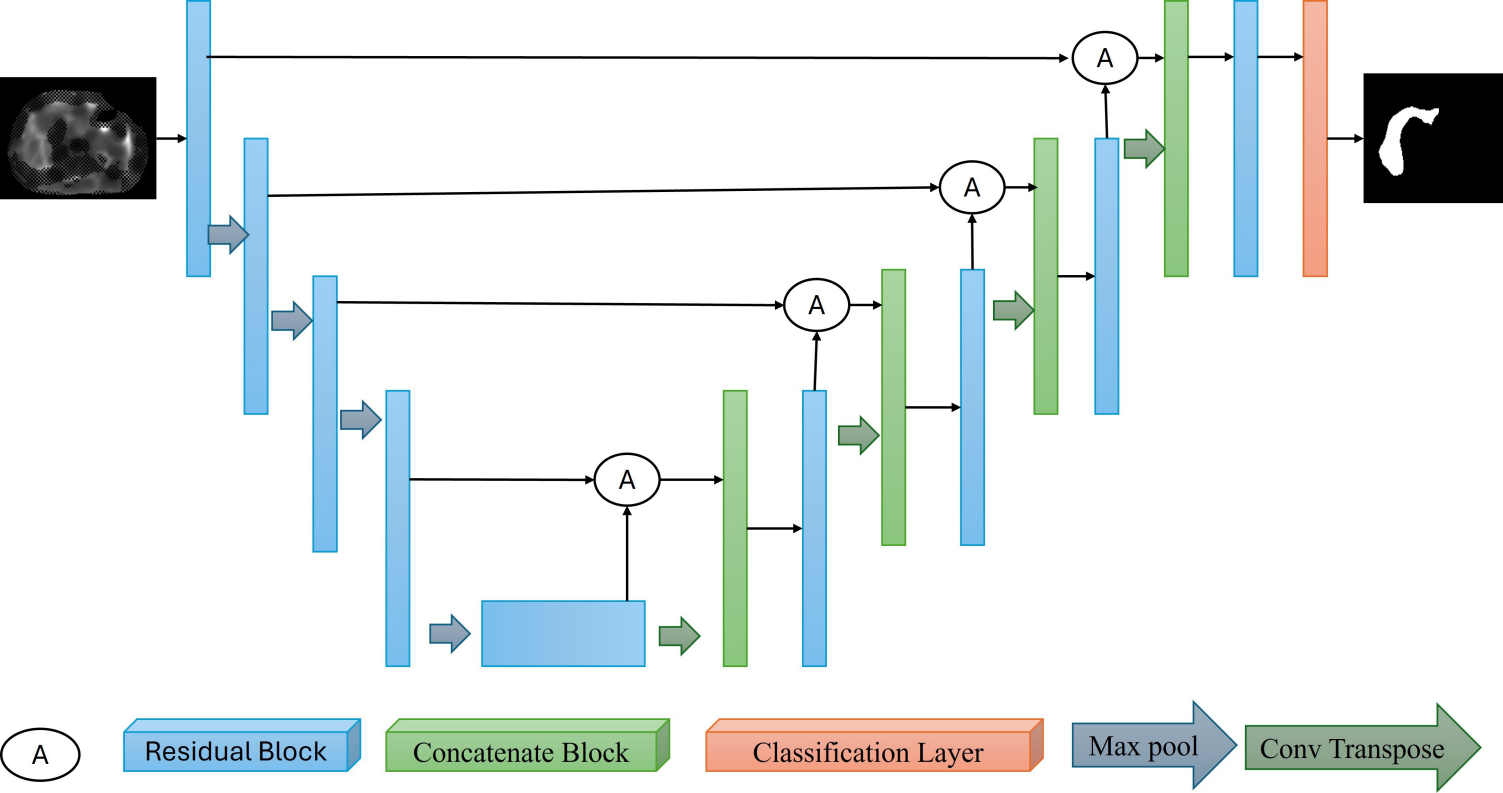
Overall architecture of the proposed UNet-ResNet50−32×4d model for automated liver segmentation and fibrosis staging using MRE confidence maps. MRE: magnetic resonance elastography.

Figure 2 illustrates the template for the auto-labeling process generated by the UNet-ResNet50−32 × 4d algorithm, a widely validated and robust architecture for medical image segmentation deep learning architecture specifically designed for semantic segmentation tasks. The auto-labeling template demonstrates the effectiveness of the model in accurately identifying and segmenting key regions of interest within the MRE confidence maps, aligning closely with expert annotations.

### Experimental Environment

All experiments in this study were conducted on a high-performance workstation equipped with an NVIDIA RTX 4090 GPU, an Intel Core i9-13900K CPU, and 128 GB of RAM. The deep learning models were implemented using PyTorch with CUDA 11.8 (torch 2.1.0+ cu118), enabling accelerated training and inference.

### Parameters Setting

Table 3 presents the optimal parameter values identified in this study through training with the UNet-ResNet50−32 × 4d algorithm, which include a learning rate of 0.005, momentum of 0.982, and weight decay of 4.457e-06.

**Table 3.** Optimal hyperparameters derived from model training.

Parameters	Value
Epoch	32
Learning rate	0.005
Momentum	0.982
Weight decay	4.457e-06

### Performance Evaluation

A comparison of the two models using the optimal parameters obtained from Table 2 indicate that the UNet-ResNet50−32 × 4d model achieved better predictive performance on the test dataset, whereas the standard U-Net model failed to produce any meaningful predictions. The average Dice coefficient, intersection over union (IoU), and F1-score for the UNet-ResNet50−32 × 4d model were 85.68%, 75.80%, and 85.68%, respectively; the corresponding values for the U-Net model were 82.59%, 75.92%, and 82.59%, respectively. The slightly lower IoU may be attributed to the presence of outliers during pixel-wise evaluation, which could have affected the overall segmentation accuracy. The segmentation performance was quantitatively assessed using the Dice coefficient, IoU, and F1-score, as shown in equations (2)–(4)


(2) $$Dice=2\vee P\cap G\vee \bar{\left|P\right|+G\vee }$$

(3) $$IoU=P\cap G\vee \bar{P\cup G\vee }$$

(4) $$F1\text{-}score=\frac{2*Precision*Recall}{Precision+Recall}$$

**Figure 3.**
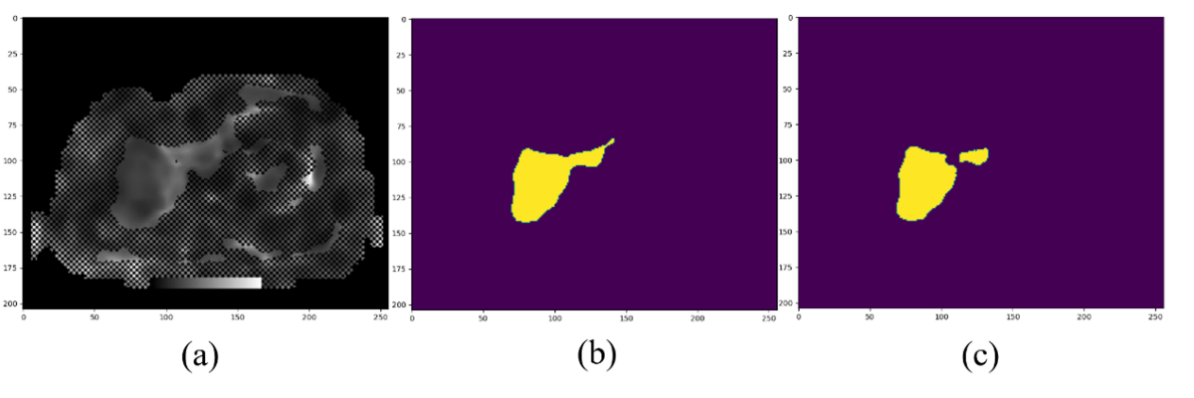
Comparative segmentation performance of conventional U-Net and UNet-ResNet50−32×4d architectures on MRE confidence maps for liver fibrosis assessment. Panel (A) shows the original MRE confidence map, while panel (B) presents the segmentation outcome generated by the conventional U-Net model, which delineates the major hepatic region but demonstrates limitations in boundary refinement and structural detail. In contrast, panel (C) depicts the result obtained using the UNet-ResNet50−32×4d model, which more accurately captures hepatic contours and structural features, highlighting its superior capability in feature extraction and region identification. MRE: magnetic resonance elastography.

Figure 3 illustrates the segmentation results for liver fibrosis imaging. Overall, the figure underscores the enhanced performance of the proposed model in automated segmentation of liver fibrosis imaging and its potential clinical applicability.

Table 4 presents the performance of the proposed UNet-ResNet50−32×4d model further evaluated across different fibrosis stages. The segmentation accuracy remained consistently high in the early stages F0-F1, with Dice scores exceeding 87% and IoU values around 78%. Performance was slightly reduced in the intermediate stage F2 and more prominently in stage F3, where Dice and IoU decreased to 80.54% and 70.49%, respectively. This decline may reflect the increased heterogeneity and irregularity of fibrosis distribution in advanced disease. Notably, the model regained relatively stable performance in stage F4, with Dice and IoU values of 84.00% and 73.14%, respectively. These findings suggest that while the model demonstrates robust segmentation across fibrosis stages, challenges remain in capturing complex tissue patterns in stage F3.

**Figure 4.**
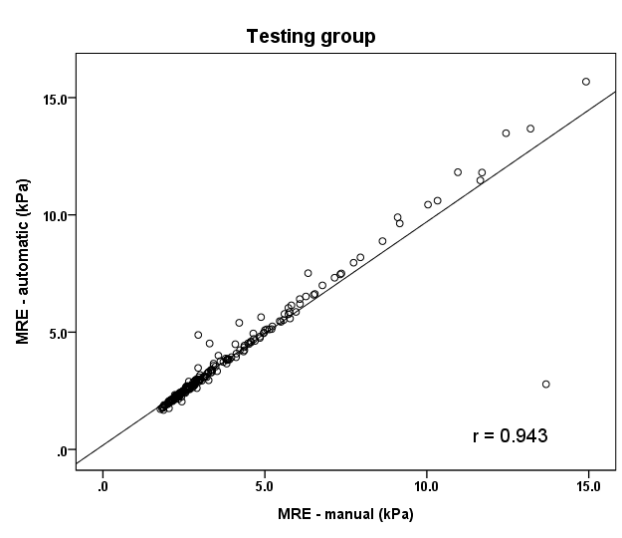
Correlation between automated segmentation–derived MRE values and manual expert measurements in the testing group. MRE: magnetic resonance electrography.

Figure 4 presents the correlation between MRE values obtained from the automated segmentation model and those measured manually by an expert gastroenterologist in the testing cohort. The analysis demonstrated a Pearson correlation coefficient of 0.943, confirming a strong positive linear relationship between the two approaches, even when applied to unseen data.

**Table 4.** Segmentation performance of the UNet-ResNet50−32×4d model across different fibrosis stages.

Fibrosis stage	Dice (%)	Intersection over union (%)	F1-score (%)
F0	87.47	78.26	87.47
F1	87.98	78.74	87.98
F2	84.11	73.85	84.11
F3	80.54	70.49	80.54
F4	84.00	73.14	84.00

## Discussion

### Principal Findings

Automated segmentation using the AI-based model for MRE measurement proved to be both reliable and effective when compared with manual segmentation. The strong correlation and reproducibility observed between the automated and manual approaches highlight the potential of this tool as a valuable aid in clinical practice. In this study, we further evaluated and compared the performance of a standard U-Net with a UNet-ResNet50−32 × 4d architecture for medical image segmentation. The findings clearly demonstrated that the UNet-ResNet50−32 × 4d model substantially outperformed the conventional U-Net in segmentation accuracy. Specifically, while the conventional U-Net achieved a Dice coefficient of 82.59% and an IoU of 72.92% on the test dataset, the proposed model further improved performance, reaching an average Dice coefficient of 85.68%, IoU of 75.80%, and F1-score of 85.68%. The slightly lower IoU relative to the Dice coefficient may be attributed to the penalization of pixel-level outliers in the union-based evaluation, which tends to disproportionately impact cases with small lesion boundaries or heterogeneous textures.

When evaluated across fibrosis stages, the model demonstrated stable performance in the early stages, with Dice scores of 87.47% and 87.98% for F0 and F1, respectively, and corresponding IoU values close to 78%. A modest decline was observed in F2 with a Dice score of 84.11% and IoU of 73.85%, with the lowest performance in F3 where the Dice score was 80.54% and IoU was 70.49%. This likely reflects the increased heterogeneity and irregularity of fibrosis distribution in advanced disease. Interestingly, the model recovered performance in F4, achieving a Dice score of 84.00% and IoU of 73.14%, suggesting that once cirrhosis is established, the fibrotic patterns may become more homogeneous and therefore easier for the model to delineate.

### Comparison to Prior Work

MRE is a highly effective non-invasive tool for evaluating liver fibrosis and serves as a valuable alternative to liver biopsy. Clinicians typically assess regions of interest using elastograms with overlaid confidence maps generated following MRI scans [31]. A previous study has investigated automated approaches for MRE measurement [32]. An early method applied intensity membership functions combined with random walker segmentation to differentiate liver tissue from surrounding structures, achieving a correlation of 0.981 with manual measurements [32]. Another study introduced volumetric segmentation using semi-automated proprietary software to evaluate liver stiffness. Their findings revealed significant differences between region of interest-based and volumetric analyses, suggesting that volumetric methods may provide better detection of heterogeneous fibrosis [33]. More recently, a CNN-based framework was applied to MRE, reporting an intraclass correlation coefficient of 0.99 between automated and manual assessments in clinical patients [19]. In this study, we applied the Unet-ResNet50 model to segment the liver on MRE confidence maps and measure liver stiffness. Our approach yielded a strong correlation of 0.943 with manual expert measurements, supporting its potential utility in clinical practice. To the best of our knowledge, this is the first study to implement a Unet-ResNet hybrid architecture for MRE segmentation and measurement. A previous study conducted in healthy volunteers reported excellent consistency and segmentation performance, with liver Dice scores reaching 0.95 [34]. This study focused on automated liver fibrosis staging (F0–F4) and extraction of MRE-derived stiffness values in kilopascals. Unlike studies limited to healthy subjects, our cohort consisted of clinical patients, in whom imaging data inherently exhibit greater heterogeneity.

### Limitations

This study has several limitations. Only gray-scale elastograms with 95% confidence maps were used for segmentation, which may have inadvertently included non-hepatic tissues such as the gallbladder fossa and large blood vessels. Despite this, the correlation between manual and automated methods remained strong. Further refinement of the segmentation process is needed to achieve more accurate anatomical delineation of the liver using the UNet-ResNet model. Key clinical data related to liver disease, such as iron levels, steatosis, and viral markers, were not collected. Severe steatosis and iron overload may interfere with MRE measurements and could not be adequately accounted for in this analysis. Histological fibrosis scores were unavailable for most participants, as liver biopsy was not routinely performed. The absence of biopsy confirmation limits the accuracy of staging, since MRE values alone may not fully capture the histopathological spectrum of fibrosis. This introduces a potential risk of mislabeling fibrosis severity, particularly in borderline cases or in patients with overlapping liver conditions. This study is subject to data imbalance, as the distribution of patients across different disease severities was uneven, which may affect the generalizability and stability of the proposed model. Although the dataset was partitioned to ensure fairness in training and testing, the uneven distribution of disease severity remains a potential source of bias. In particular, when the model is trained on patients with more advanced disease, its application to cohorts with milder disease may result in an overestimation of fibrosis severity and diminished sensitivity to early-stage changes. Conversely, if a model is optimized for mild cases, it may underperform in advanced disease populations, resulting in systematic misclassification. These imbalances highlight the need for future studies that incorporate paired biopsy and imaging data, along with balanced cohorts across different severities, to validate the robustness and clinical applicability of automated MRE-based staging.

### Future Directions

Future investigations will need to progress beyond algorithmic refinement and incorporate the expansion of patient cohorts, thereby increasing statistical power and enhancing the robustness of predictive models. Validation through multi-center studies will establish reproducibility across institutions, imaging protocols, and heterogeneous patient demographics. In parallel, the integration of multimodal information, including complementary imaging techniques, clinical parameters, and biomarker profiles, will strengthen both predictive accuracy and clinical relevance. The inclusion of expert annotations from multiple specialists, encompassing hepatologists, radiologists, pathologists, and other domain experts, will reduce inter-observer variability and further reinforce the clinical validity of model outputs. These efforts will enable broader generalizability across diverse populations and disease severities, ultimately supporting translation into routine clinical practice.

### Conclusions

Early and accurate assessment of liver fibrosis is essential for enabling timely diagnosis and intervention. In this study, we developed a U-Net-ResNet50−32 × 4d model to predict the severity of liver fibrosis. The model achieved correlation coefficients above 0.9 in both training and validation cohorts and reached an Dice score of 85.68%, demonstrating strong potential to support accurate fibrosis staging in clinical practice. Importantly, liver fibrosis staging currently lacks a universally accepted gold standard. While histology remains the traditional reference, it is invasive, limited by sampling error, and not always available. Our findings suggest that automated MRE-based methods may provide a reliable non-invasive alternative, although the absence of biopsy confirmation introduces potential uncertainties in staging accuracy.

The strong performance of our model highlights its practical value and potential to improve efficiency in clinical decision-making. Future research should validate these results in larger, multi-center cohorts, integrate complementary imaging modalities and clinical biomarkers to further strengthen predictive power, and explore real-time deployment within radiology workflows to maximize clinical applicability.

## Abbreviations

AI: artificial intelligence
CNN: convolutional neural network
CT: computed tomography
DICOM: Digital Imaging and Communications in Medicine
IoU: intersection over union
MRE: magnetic resonance elastography
MRI: magnetic resonance imaging
